# *Capparis spinosa* Fruit Ethanol Extracts Exert Different Effects on the Maturation of Dendritic Cells

**DOI:** 10.3390/molecules22010097

**Published:** 2017-01-07

**Authors:** Azeguli Hamuti, Jinyu Li, Fangfang Zhou, Adila Aipire, Ji Ma, Jianhua Yang, Jinyao Li

**Affiliations:** 1 Xinjiang Key Laboratory of Biological Resources and Genetic Engineering, College of Life Science and Technology, Xinjiang University, 666 Shengli Road, Urumqi 830046, China; 13070411128@163.com (A.H.); lijinyu234@163.com (J.L.); 15199131636@163.com (F.Z.); kaskas999@163.com (A.A.); majiuci@xju.edu.cn (J.M.); jianhuay@bcm.edu (J.Y.); 2 Texas Children’s Cancer Center, Department of Pediatrics, Dan L. Duncan Cancer Center, Baylor College of Medicine, Houston, TX 77030, USA

**Keywords:** *Capparis spinosa*, dendritic cell, maturation, cytokine production

## Abstract

*Capparis spinosa* L. (*C. spinosa*) has been used as food and traditional medicine and shows anti-inflammatory and anti-oxidant activities. Here, we prepared the *C. spinosa* fruit ethanol extracts (CSEs) using different procedures and investigated the effects of CSE on the maturation of mouse bone marrow-derived dendritic cells (DCs) in the absence or presence of lipopolysaccharide (LPS). DC maturation and cytokine production were detected by flow cytometry and ELISA, respectively. We obtained three different CSEs and dissolved in water or DMSO, named CSE2W, CSEMW, CSE3W, CSE2D, CSEMD, and CSE3D, respectively. These CSEs showed different effects on DC maturation. CSEMW and CSEMD significantly increased the expressions of CD40, CD80, and CD86, in a dose-dependent manner. CSE2W and CSE2D also showed a modest effect on DC maturation, which enhanced the expression of CD40. CSE3W and CSE3D did not change DC maturation but suppressed LPS-induced DC maturation characterized by the decreased levels of CD40 and CD80. CSE3W and CSE3D also significantly inhibited the secretions of IL-12p40, IL-6, IL-1β, and TNF-α induced by LPS. CSE3W further increased the level of IL-10 induced by LPS. Moreover, CSE3D suppressed LPS-induced DC maturation in vivo, which decreased the expressions of CD40 and CD80. These results suggested that CSE3W and CSE3D might be used to treat inflammatory diseases.

## 1. Introduction

Traditional Chinese medicines (TCM) have been used to treat various kinds of diseases for a long history. Accumulating evidence has shown that TCM exerts immunoregulatory effects to prevent or treat infection or autoimmune diseases [1,2]. TCM could promote the maturation of dendritic cells (DCs) to enhance immune responses against infection, or suppress DC maturation to inhibit immune responses against inflammation or autoimmune diseases [1,3]. *Capparis spinosa* L. is a kind of TCM and has been used to treat gastro-intestinal problems, rheumatism, gout, diabetes, inflammation, kidney, and liver diseases [4,5,6,7]. In China, *C. spinosa* (also named wild watermelon) mainly distributed in Xinjiang Uygur Autonomous Region, which is a kind of sand binding plant and its buds, leaves, roots, and fruits have been used in traditional Uighur Medicine to treat gout and rheumatoid arthritis for a long time [8]. Recently, many studies have been reported that *C. spinosa* contains a lot of biochemical compounds including flavonoids, alkaloids, polyphenols, saponins, terpenoids, lectin, essential oils, glycosinolate, and glycosides [6,9,10,11,12], which exhibited a broad range of activities such as anti-inflammatory [13,14,15,16], anti-viral [17,18], anti-allergeric [19], anti-arthritic [20], anti-tumor [9,17,21], anti-oxidant [22,23,24,25,26], anti-nociceptive [27,28], anti-diabetic [4,29], anti-hepatotoxic [30], anti-hyperglycemic [31], hypolipidemic [32], and immunomodulatory [18,33]. However, the effect of *C. spinosa* on the maturation and cytokine production of DCs is still elusive.

DCs are professional antigen presenting cells and play a pivotal role in the immune system, which link the innate and adaptive immune responses. The activation status of DCs, including maturation and cytokine secretion, determined the development of CD4^+^ helper T (Th) cell subsets that helped CD8^+^ T cells to generate cytotoxic T lymphocytes and B cells to produce antibodies [34,35]. Therefore, immune responses including inflammation could be modulated through the regulation of DC activation status. In order to investigate whether *C. spinosa* can affect the DC activation status to exert its some pharmacological effects, such as anti-inflammatory, anti-allergic, and immunomodulatory, we prepared *C. spinosa* fruit ethanol extracts (CSEs) and detected their effects on the maturation and cytokine production of DCs, especially in the presence of lipopolysaccharide (LPS).

## 2. Results

### 2.1. The Preparation of CSEs and Their Effect on DC Viability 

**Figure 1:**
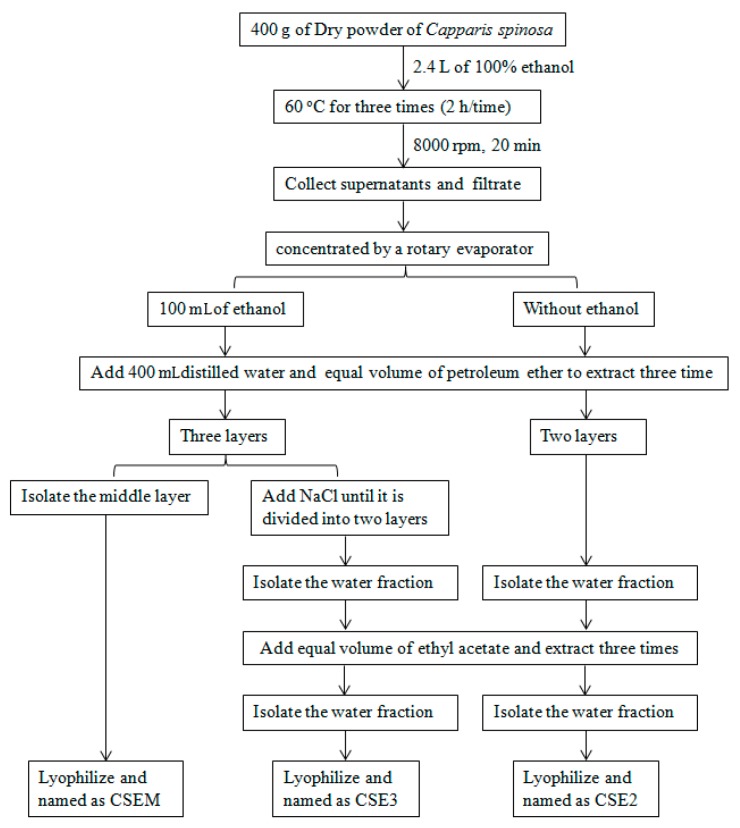
The extraction procedures for CSE2, CSEM, and CSE3.

CSEs were prepared using ethanol extract following the extraction with petroleum ether and ethyl acetate according to previous description [36]. To separate the components with inhibitory activity from other components with stimulative activity on DC maturation, we optimized the procedures through changing the content of ethanol during evaporation and adding NaCl after petroleum ether extraction (Figure 1). We collected these fractions and named them CSE2, CSEM, and CSE3. These CSEs were dissolved in distilled water or DMSO at 200 mg/mL and named CSE2W, CSEMW, and CSE3W; or CSE2D, CSEMD and CSE3D, respectively. The contents of flavonoids and polysaccharides were determined by AlCl3-KAC and anthrone-sulfuric acid method [37,38], respectively. The concentrations of polysaccharides and flavonoids in CSEs were shown in Table 1.

**Figure 2:**
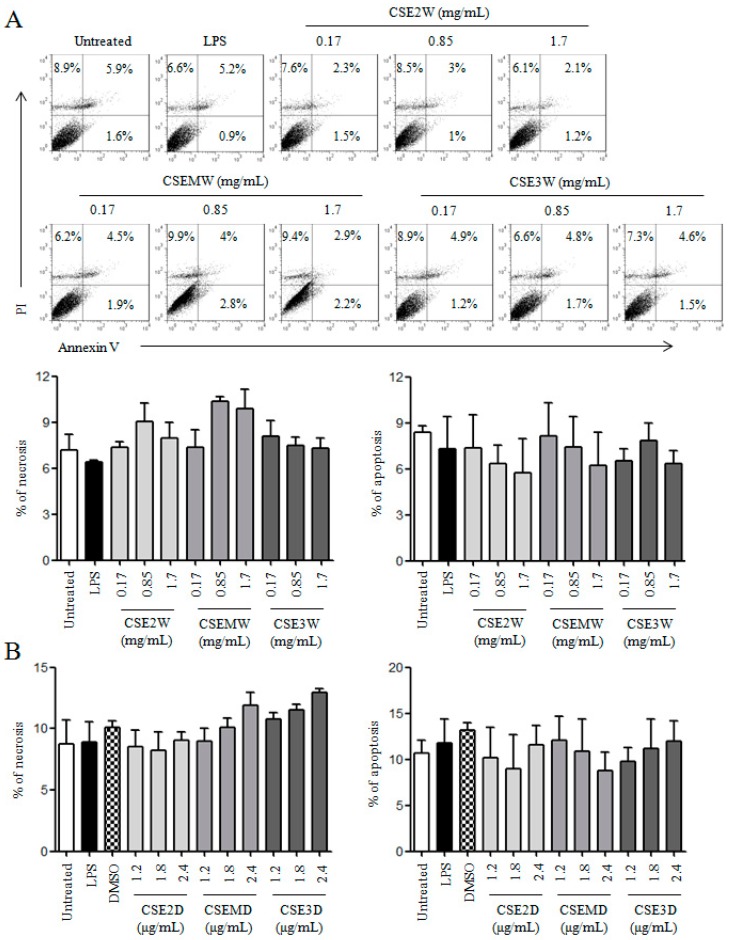
The effect of CSEs on DC viability. Mouse bone marrow-derived DCs were treated with different concentrations of polysaccharides in CSE2W, CSEMW, and CSE3W (**A**) or flavonoids in CSE2D, CSEMD, and CSE3D (**B**). After 12 h, cells were collected and stained with Annexin V/PI kit. The representative dot plots are shown in upper panels. The summary of necrotic and apoptotic DCs are shown in lower panels.

To detect the effect of CSEs on DC viability, different concentrations of CSE2W, CSEMW, and CSE3W according to their polysaccharide contents (0.17, 0.85, and 1.7 mg/mL) or CSE2D, CSEMD, and CSE3D according to their flavonoid contents (1.2, 1.8, and 2.4 μg/mL) were used to treat DCs. After 12 h, DCs were collected and stained with PI/Annexin V kit. Samples were analyzed by flow cytometry. The results showed that all CSEs in selected concentrations did not significantly change the frequencies of necrotic (PI^+^Annexin V^−^) and apoptotic (PI^−^Annexin V^+^ and PI^+^Annexin V^+^) DCs (Figure 2), suggesting that CSEs did not dampen the viability of DCs.

### 2.2. The Effect of Polysaccharides in CSEs on DC Maturation and Cytokine Production

**Figure 3:**
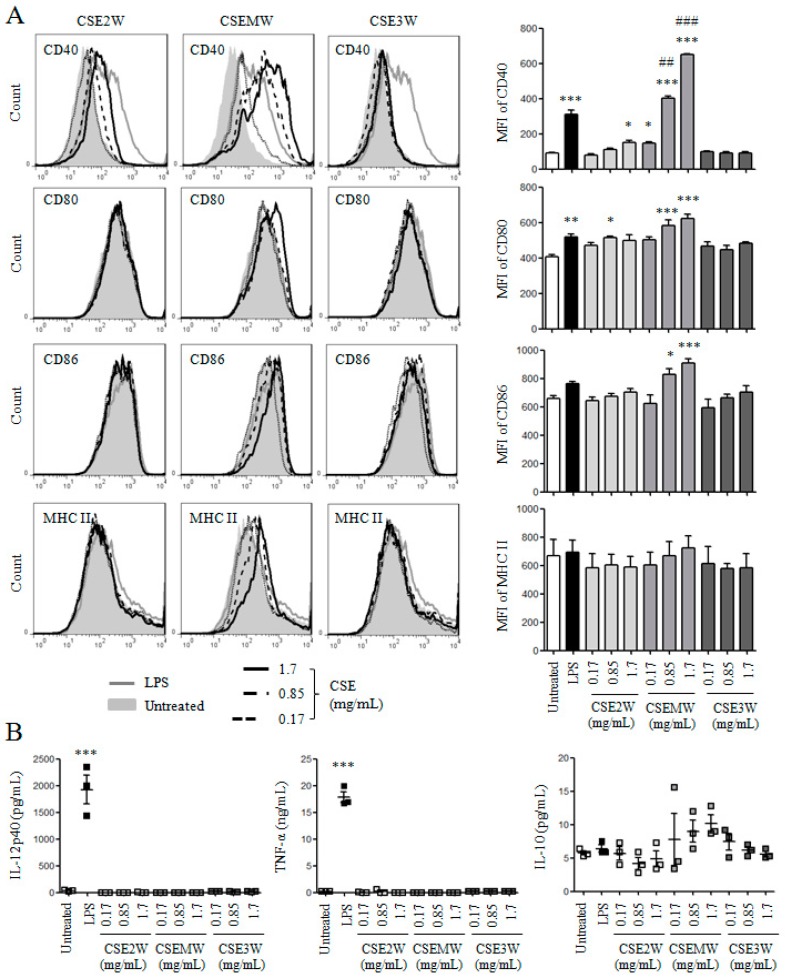
The effect of polysaccharides in CSE2W, CSEMW, and CSE3W on DC maturation. DCs were treated with different concentrations of polysaccharides in CSE2W, CSEMW, and CSE3W for 12 h. (**A**) Cells were collected to analyze the expressions of CD40, CD80, CD86, and MHC II by flow cytometry. MFI of these molecules is shown; (**B**) The supernatants were collected to detect the secretions of IL-12p40, IL-10, and TNF-α by ELISA. The concentrations of IL-12p40, IL-10, and TNF-α are shown. Data are from four independent experiments and analyzed by ANOVA. * *p* < 0.05; ** *p* < 0.01; *** *p* < 0.001 compared to untreated DCs. ## *p* < 0.01; ### *p* < 0.001 compared to LPS treated DCs.

Several studies including ours have been reported that polysaccharides from herbal medicines can promote DC maturation and cytokine production [39,40,41,42]. The effect of polysaccharides in CSE2W, CSEMW, and CSE3W on DC maturation was investigated. After treatment with different polysaccharide concentrations (0.17, 0.85, and 1.7 mg/mL) of CSEs, the expressions of CD40, CD80, CD86, and MHC II on DCs were detected by flow cytometry (Figure 3A). We observed that CSEMW significantly increased the expressions of CD40, CD80, and CD86, in a dose-dependent manner, which is even higher than that induced by LPS. The highest and middle dose of CSE2W also significantly increased the expressions of CD40 and CD80, respectively, although the level is dramatically lower than that induced by CSEMW. However, CSE3W did not increase the expressions of all these molecules. The levels of IL-12p40, TNF-α, and IL-10 in supernatants of the above samples were measured by ELISA. As shown in Figure 3B, LPS induced high levels of IL-12p40 and TNF-α but all CSEs dissolved in water did not increase the secretions of IL-12p40 and TNF-α. Compared to the untreated group, the level of IL-10 had no significant change upon the treatment of CSEs. These results indicate that polysaccharides in CSEs have different effects on DC maturation.

### 2.3. The Effect of Flavonoids in CSEs on DC Maturation and Cytokine Production

**Figure 4:**
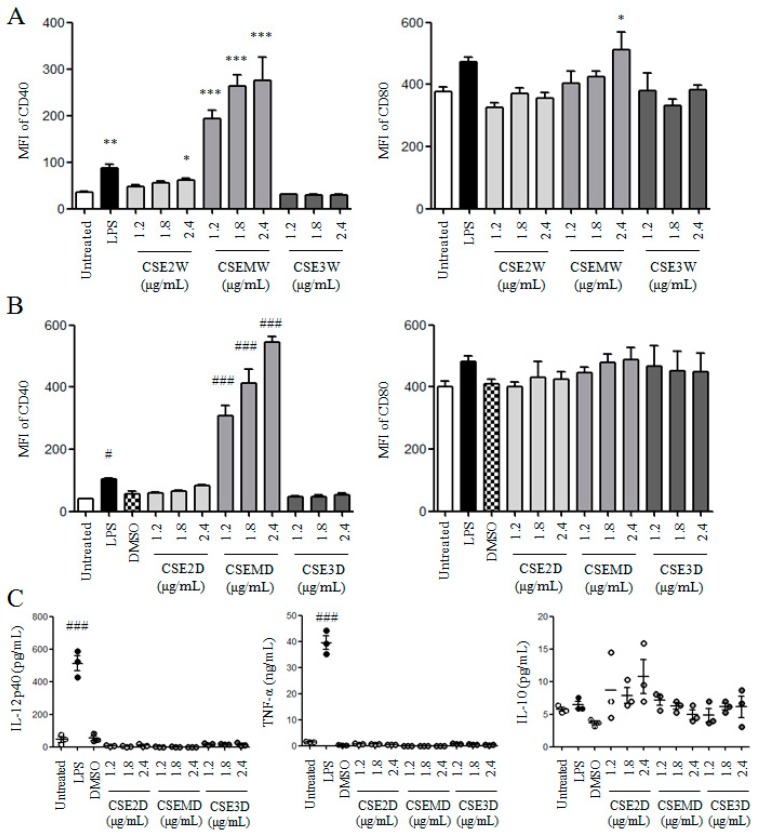
The effect of flavonoids in CSEs on DC maturation. DCs were treated with different concentrations of flavonoids in CSEs for 12 h. Cells were collected to analyze the expressions of CD40 and CD80 by flow cytometry. (**A**) MFI of CD40 and CD80 upon CSE2W, CSEMW and CSE3W treatment; (**B**) MFI of CD40 and CD80 upon CSE2D, CSEMD, and CSE3D treatment; (**C**) The supernatants were collected to detect the levels of IL-12p40, IL-10, and TNF-α by ELISA after CSE2D, CSEMD, and CSE3D treatment. The concentrations of IL-12p40, IL-10, and TNF-α are shown. Data are from four independent experiments and analyzed by ANOVA. * *p* < 0.05; ** *p* < 0.01; *** *p* < 0.001 compared to untreated DCs. # *p* < 0.05; ### *p* < 0.001 compared to DMSO treated DCs.

It has been shown that flavonoids in *C. spinosa* have anti-inflammatory activity [14,16]. Therefore, we detected the effect of flavonoids in CSEs on DC maturation and cytokine production. Firstly, different flavonoid concentrations (1.2, 1.8, and 2.4 μg/mL) of CSE2W, CSEMW, and CSE3W were used to treat DCs. After 12 h, the expressions of CD40 and CD80 on DCs were measured by flow cytometry. Similar with Figure 3A, CSEMW dose-dependently enhanced the expressions of CD40 and CD80 and the stimulatory activity was higher than that of LPS. CSE2W also significantly enhanced the expression of CD40 but not for CD80. CSE3W still did not enhance the expressions of these molecules (Figure 4A). Secondly, the same flavonoid concentrations of CSE2D, CSEMD, and CSE3D were used to treat DCs for 12 h. Similar results were obtained compared with CSE2W, CSEMW, and CSE3W contained the same concentrations of flavonoids (Figure 4B). All CSEs dissolved in DMSO did not increase the production of IL-12p40, TNF-α, and IL-10 (Figure 4C).

### 2.4. CSE3D and CSE3W Suppressed DC Maturation and Cytokine Production Induced by LPS

**Figure 5:**
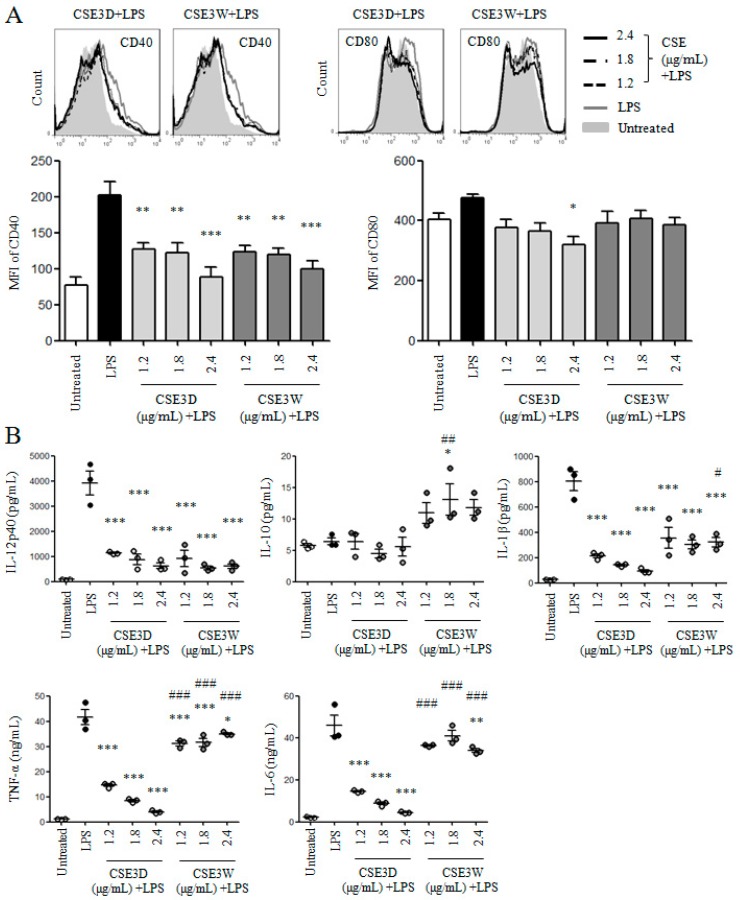
CSE3D and CSE3W suppressed DC maturation and cytokine production induced by LPS. DCs were treated with different concentrations of flavonoids in CSE3D and CSE3W in the presence of LPS for 12 h. (**A**) Cells were collected to analyze the expressions of CD40 and CD80 by flow cytometry. MFI of CD40 and CD80 is shown; (**B**) The supernatants were collected to detect the levels of IL-12p40, IL-10, IL-6, IL-1β, and TNF-α by ELISA. The concentrations of these cytokines are shown. Data are from three independent experiments and analyzed by ANOVA. * *p* < 0.05; ** *p* < 0.01; *** *p* < 0.001 compared to LPS treated DCs. # *p* < 0.05; ## *p* < 0.01; ### *p* < 0.001 compared to CSE3D + LPS treated DCs.

The above results showed that both CSE3D and CSE3W did not promote DC maturation and cytokine production. We wondered whether CSE3D and CSE3W could suppress DC maturation induced by LPS. DCs were treated with different flavonoid concentrations (1.2, 1.8, and 2.4 μg/mL) of CSE3D and CSE3W in the presence of LPS. After 12 h, the stained samples were analyzed by flow cytometry. The results showed that both CSE3D and CSE3W significantly suppressed the expression of CD40 induced by LPS, in a dose-dependent manner. CSE3D also significantly suppressed the expression of CD80 induced by LPS but not for CSE3W (Figure 5A).

The levels of anti-inflammatory IL-10 and pro-inflammatory cytokines including IL-12p40, IL-6, IL-1β, and TNF-α in supernatants of the above samples were determined by ELISA. We found that the levels of all these pro-inflammatory cytokines induced by LPS were significantly decreased by CSE3D and CSE3W (Figure 5B). Moreover, the inhibitory activity of CSE3D is stronger than that of CSE3W. However, the level of IL-10 induced by LPS was significantly increased by 1.8 μg/mL CSE3W. These results suggested that CSE3D and CSE3W could suppress DC maturation and pro-inflammatory cytokine production induced by LPS.

### 2.5. CSE3D Suppressed DC Maturation Induced by LPS In Vivo

**Figure 6:**
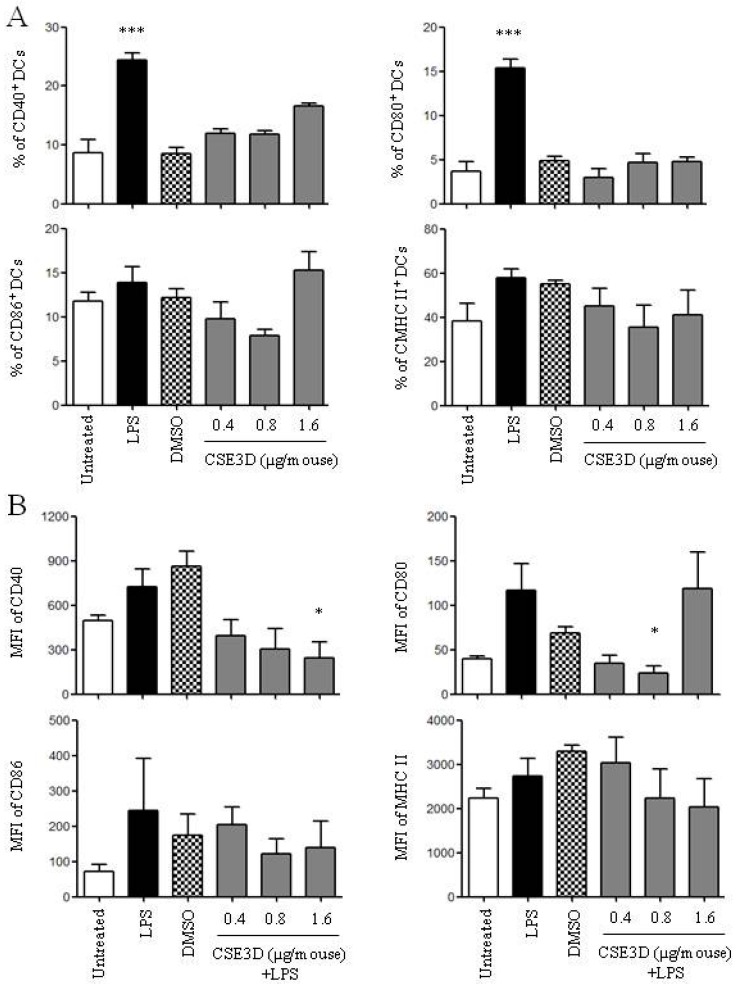
CSE3D suppressed DC maturation induced by LPS in vivo. Mice were injected with different concentrations of flavonoids in CSE3D in the absence (**A**) or presence (**B**) of LPS by footpads. After 24 h, lymphocytes were isolated to analyze DC maturation. CD11c^+^ cells were gated to analyze the expressions of CD40, CD80, CD86, and MHC II. MFI of these molecules is shown. *** *p* < 0.001 compared to untreated mice. * *p* < 0.05 compared to LPS treated mice.

The in vivo effect of CSE3D on DC maturation in the absence or presence of LPS was further investigated. BALB/c mice were injected with different flavonoid concentrations (0.4, 0.8, and 1.6 μg/mouse) of CSE3D by footpads. Mice injected with LPS alone were used as a positive control. After 24 h, popliteal lymph nodes were isolated and lymphocytes were used to analyze the expressions of CD40, CD80, CD86, and MHC II on DCs (CD11c^+^) by flow cytometry. The results showed that LPS significantly increased the expressions of CD40 and CD80 but CSE3D did not significantly change the expressions of all these molecules on DCs in vivo (Figure 6A).

In order to detect the effect of CSE3D on DC maturation in vivo in the presence of LPS, mice were injected with different flavonoid concentrations (0.4, 0.8, and 1.6 μg/mouse) of CSE3D together with 100 ng/mouse of LPS by footpads. After 24 h, lymphocytes in popliteal lymph nodes were isolated to analyze DC maturation by flow cytometry. We observed that 1.6 and 0.8 μg/mouse of CSE3D significantly inhibited the expressions of CD40 and CD80 induced by LPS, respectively (Figure 6B). The expressions of CD86 and MHC II induced by LPS were also decreased although the difference was not significant. These results indicated that CSE3D could suppress DC maturation both in vitro and in vivo.

## 3. Discussion

We prepared the ethanol extracts of *C. spinosa* using different procedures and detected the effect of these extracts on DC maturation both in vitro and in vivo. We found that CSE2W, CSE2D, CSEMW, and CSEMD could promote DC maturation but CSE3W and CSE3D did not promote DC maturation. Interestingly, CSE3W and CSE3D suppressed DC maturation and pro-inflammatory cytokine production induced by LPS in vitro. CSE3D also suppressed DC maturation in vivo.

It has been reported that *C. spinosa* contained lots of components and showed various pharmacological activities including anti-inflammatory [6]. Several studies have been shown that flavonoids of *C. spinosa* are contributed to the anti-inflammatory activity [14,16]. DCs are the key regulators for the homeostasis of immune system and their changes in distribution, function, and cytokine production are closely correlated with autoimmune and inflammatory disorders [43,44]. Here, we found that CSEs contained same concentration of flavonoids exhibited different effects on DC maturation. CSEM and CSE2 promote DC maturation but CSE3 did not. Moreover, CSE3 suppressed DC maturation and pro-inflammatory cytokine production including IL-12, IL-6, IL-1β, and TNF-α induced by LPS. The results indicated that CSEs might contain different components that resulted in different effects on DC maturation. The flavonoids in CSE3 might be the critical components that caused the anti-inflammatory activity to suppress cytokine production of DCs induced by LPS. Therefore, other components in CSEM and CSE2 or different flavonoids with them in CSE3 might promote DC maturation.

Some polysaccharides of herbal medicine could promote DC maturation and cytokine production [39,40,41,42]. We observed that CSEs contained high levels of polysaccharides. We used the same concentrations of polysaccharides from CSEs to treat DCs and found that CSEM had highest stimulatory activity to promote DC maturation, followed by CSE2. However, CSE3 did not promote DC maturation. The ratios of polysaccharides/flavonoids are 376, 85, and 368 in CSE2D, CSEMD, and CSE3D (Table 1). These results suggested that polysaccharides might not be the active components to promote DC maturation or different polysaccharides in CSEs had different effects on DC maturation. Bilen et al. [33] reported that caper methanolic extract stimulates innate immunity in rainbow trout, which elevated the levels of TNF-α, IL-12p40, and IL-10 and increased phagocytic activity. In a future study, we will purify the active components that suppress or enhance DC maturation.

Taken together, CSE3 could suppress DC maturation and cytokine production induced by LPS both in vitro and in vivo, suggesting that CSE3 might be used as anti-inflammatory agent to treat arthritic and allergic diseases.

## 4. Materials and Methods

### 4.1. Preparation of *C. spinosa* Ethanol Extracts

The *C. spinosa* ethanol extracts were prepared using the procedures that were shown in Figure 1. Generally, the dried fruit of *C. spinosa* was crushed into powder. 400 g of powder was extracted using 2.4 L of absolute ethanol at 60 °C three times (2 h/time). The extracts were collected, filtrated, and concentrated by a rotary evaporator. For CSE3 and CSEM, the ethanol extract was concentrated to 100 mL, 400 mL of distilled water was added and then extracted with petroleum ether three times. The extract was divided into three layers when it was extracted with petroleum ether. On the one hand, the middle layer was isolated and lyophilized and named as CSEM. On the other hand, the extract was treated with NaCl until it was divided into two layers. The water fraction was isolated and extracted with ethyl acetate three times, then the water fraction was concentrated by a rotary evaporator. During evaporation, crystallized NaCl was timely removed. Finally, this fraction was lyophilized and named as CSE3. For CSE2, the ethanol extract was concentrated to ethanol-free, 400 mL of distilled water was added and then extracted with petroleum ether three times. The extract was divided into two layers. The water fraction was isolated and extracted with ethyl acetate three times, then the water fraction was lyophilized and named CSE2. These CSEs were dissolved in distilled water or DMSO (Sigma-Aldrich, St. Louis, MO, USA) at 200 mg/mL and named CSE2W, CSEMW, and CSE3W; or CSE2D, CSEMD, and CSE3D, respectively.

The contents of flavonoids and polysaccharides were determined by AlCl3-KAC and anthrone-sulfuric acid method, respectively.

### 4.2. Mice and Injection

BALB/c mice were, aged 6–8 weeks, purchased from the Beijing laboratory animal research center (Beijing, China). Mice were housed in a temperature-controlled, light-cycled animal facility of Xinjiang University. All animal experiments were approved by the Committee on the Ethics of Animal Experiments of Xinjiang Key Laboratory of Biological Resources and Genetic Engineering and carried out under the guidelines of the Animal Care and Use Committee of College of Life Science and Technology, Xinjiang University.

Mice (3 mice/group) were injected with different concentrations (0.4, 0.8, and 1.6 μg/mouse) of flavonoids in CSE3D or LPS (100 ng/mouse) by both sides of footpads (25 μL/footpad). After 24 h, popliteal lymph nodes were isolated to analyze DC maturation by flow cytometry.

### 4.3. The Generation of Dendritic Cell and CSE Treatment

Immature DCs were induced from bone marrow cells of BALB/c mice in the presence of 20 ng/mL of GM-CSF (Peprotech) according to the previous protocol [45]. These cells were collected on day 7 and treated with different concentrations of CSEs in the absence or presence of 20 ng/mL LPS (Sigma-Aldrich). LPS treatment was used as a positive control.

### 4.4. Enzyme-Linked Immunosorbent Assay (ELISA)

Upon CSEs or LPS treatment, the levels of IL-12p40, IL-10, IL-6, IL-1β, and TNF-α in supernatants of the above samples were measured by ELISA kit according to the manufacturer’s instruction (Elabscience, Wuhan, China). The OD values at 450 nm were obtained using an ELISA plate reader (Bio-Rad, Hercules, CA, USA) and the concentrations of cytokines were calculated according to the standard curve.

### 4.5. Flow Cytometry

Annexin V/PI staining kit (Shanghai Yeasen Biotechnology Co., Ltd., Shanghai, China) was used to detect the viability of DCs upon CSE treatment and performed according to the manufacturer’s instruction. For analysis of DC maturation, cell surface staining was performed using the mAbs (BD Biosciences, San Jose, CA, USA): PE-CD11c, APC-CD40, FITC-CD80, APC-CD11c, FITC-CD86, or PE-MHC II. All samples were collected on FACSCalibur (BD Biosciences) and the data were analyzed by the FlowJo platform (Tree Star, Inc., Ashland, OR, USA). CD11c^+^ cells were gated to analyze the expressions of CD40, CD80, CD86, and MHC II on DCs in all experiments.

### 4.6. Statistical Analysis

Statistical analysis was done by one-way analysis of variance (ANOVA). *p* < 0.05 was considered to be statistically significant.

## Figures and Tables

**Table 1:** Contents of flavonoids and polysaccharides in *Capparis spinosa* extracts.

Extracts	Polysaccharides (mg/mL)	Flavonoids (mg/mL)	Ratios of Polysaccharides/Flavonoids
CSE2W	115.58	0.34	340
CSE2D	97.87	0.26	376
CSEMW	67.17	0.46	146
CSEMD	66.91	0.79	85
CSE3W	133.23	0.2	666
CSE3D	58.81	0.16	368
